# Modulation of Glucagon Receptor Pharmacology by Receptor Activity-modifying Protein-2 (RAMP2)*

**DOI:** 10.1074/jbc.M114.624601

**Published:** 2015-07-21

**Authors:** Cathryn Weston, Jing Lu, Naichang Li, Kerry Barkan, Gareth O. Richards, David J. Roberts, Timothy M. Skerry, David Poyner, Meenakshi Pardamwar, Christopher A. Reynolds, Simon J. Dowell, Gary B. Willars, Graham Ladds

**Affiliations:** From the ‡ Division of Biomedical Cell Biology, Warwick Medical School, University of Warwick, Coventry CV4 7AL, United Kingdom,; the § Department of Cell Physiology and Pharmacology, University of Leicester, Leicester LE1 9HN, United Kingdom,; the ¶ Mellanby Centre for Bone Research, Department of Human Metabolism, University of Sheffield, Sheffield S10 2RX, United Kingdom,; the ‖ School of Life Sciences, Aston University, Aston Triangle, Birmingham B4 7ET, United Kingdom,; the ** School of Biological Sciences, University of Essex, Wivenhoe Park, Colchester CO4 3SQ, United Kingdom, and; the ‡‡ Department of Biological Sciences, Molecular Discovery Research, GlaxoSmithKline, Hertfordshire SG1 2NY, United Kingdom, and; the §§ Department of Pharmacology, University of Cambridge, Tennis Court Road, Cambridge CB2 1PD, United Kingdom

**Keywords:** G protein-coupled receptor (GPCR), glucagon, pharmacology, signal transduction, type 2 diabetes, glucagon receptor, glucagon-like peptide-1, receptor activity-modifying proteins (RAMPs), signal bias

## Abstract

The glucagon and glucagon-like peptide-1 (GLP-1) receptors play important, opposing roles in regulating blood glucose levels. Consequently, these receptors have been identified as targets for novel diabetes treatments. However, drugs acting at the GLP-1 receptor, although having clinical efficacy, have been associated with severe adverse side-effects, and targeting of the glucagon receptor has yet to be successful. Here we use a combination of yeast reporter assays and mammalian systems to provide a more complete understanding of glucagon receptor signaling, considering the effect of multiple ligands, association with the receptor-interacting protein receptor activity-modifying protein-2 (RAMP2), and the role of individual G protein α-subunits. We demonstrate that RAMP2 alters both ligand selectivity and G protein preference of the glucagon receptor. Importantly, we also uncover novel cross-reactivity of therapeutically used GLP-1 receptor ligands at the glucagon receptor that is abolished by RAMP2 interaction. This study reveals the glucagon receptor as a previously unidentified target for GLP-1 receptor agonists and highlights a role for RAMP2 in regulating its pharmacology. Such previously unrecognized functions of RAMPs highlight the need to consider all receptor-interacting proteins in future drug development.

## Teaser

**Background:** The glucagon and glucagon-like peptide-1 (GLP-1) receptors are important targets for treating type 2 diabetes.

**Results:** We describe novel glucagon receptor pharmacology, through interaction with the receptor activity-modifying protein-2 (RAMP2).

**Conclusion:** RAMP2 regulates both ligand binding and G protein selectivity of the glucagon receptor.

**Significance:** The effect of RAMP2 should be considered when designing anti-diabetic treatments.

## Introduction

Glucagon, released from pancreatic α-cells, is generally considered as a counterregulatory hormone to insulin (1). It acts via the glucagon receptor (GCGR),^3^ a G protein-coupled receptor (GPCR), to stimulate the release of glucose from the liver into the blood (2). High blood glucose levels, caused by an imbalance in plasma levels of insulin and glucagon, are characteristic of type 2 diabetes. The stimulation of insulin release, via activation of the glucagon-like peptide-1 (GLP-1) receptor, (a GPCR closely related to the GCGR) has been the focus of intense research, resulting in the approval of several drugs (3). Due to the role that excess GCGR activity may play in the development of hyperglycemia, compounds acting at this receptor (to antagonize signaling) are also highly sought after (4). However, despite several years of research with many patents for small molecule compounds filed (5), there are currently no successful drugs approved for clinical use.

The GCGR mediates its effects predominantly through the generation of intracellular cAMP via coupling to heterotrimeric G proteins containing Gα_s_ (6). However, the GCGR can adopt multiple active conformations, thereby regulating other downstream pathways, including members of the inhibitory Gα_i_ family (7), which antagonize cAMP production. Distinct cellular outcomes can therefore be driven, through the stabilization of different receptor conformations (8) by interacting molecules (including ligands, G proteins, and accessory proteins). Understanding how each of the interactions influences GCGR signaling could lead to the development of ligands that selectively engage therapeutically beneficial pathways, thereby producing more efficacious drugs with greater specificity and fewer side effects. To this end, we sought to investigate the effect of association with a class of interacting proteins, the receptor activity-modifying proteins (RAMPs), on GCGR pharmacology. RAMPs have previously been shown to modulate the ligand preference for other family B GPCRs (9, 10). However, no comprehensive study of their role in ligand and G protein selection has thus far been undertaken.

The three RAMPs (RAMP1, RAMP2, and RAMP3) were first identified as being essential components of the receptors for calcitonin gene-related peptide (CGRP) and adrenomedullin (AM) (11). In addition to this well characterized interaction with the calcitonin-like-related receptor (CLR), where they are required to facilitate trafficking to the cell surface, RAMPs also associate with other family B GPCRs, including the calcitonin receptor, to modulate ligand and G protein selection (12, 13) the vasoactive intestinal polypeptide/pituitary AC-activating peptide 1 (VPAC1) receptor, and the GCGR (9, 10).

Although the GCGR interacts with RAMP2 (9), the impact of this association on signaling and physiology has not been determined. Here we report that RAMP2 association significantly alters the pharmacology of all GCGR ligands. This modulation is dependent upon the activating ligand and the downstream G protein pathway. Furthermore, we show that ligands of the GLP-1 receptor are able to act as agonists at the GCGR but that this is abolished by RAMP2 interaction. These results demonstrate the complex interplay between the ligand, the GCGR, and the RAMP that alters the signaling bias of the receptor. Importantly, the study highlights the GCGR as a novel and potentially significant target for drugs designed to provide agonism at the GLP-1 receptor, which may now be tractable if the role of RAMP2 is considered. The outcome of our work could therefore impact upon new or improved drugs to treat type 2 diabetes.

## Experimental Procedures

### 

#### 

##### Peptides

Glucagon, oxytomodulin, and GLP-1(7–36) amide were synthesized by Alta Biosciences (University of Birmingham, Birmingham, UK) and prepared as 1 mm stocks in water. Liraglutide, exenatide, and lixisenatide were supplied by George Eliot Hospital NHS Trust (Nuneaton, UK). CGRP and AM were purchased from BACHEM (Bubendorf, Switzerland) and made to 1 mm stocks in water. The radioligand, [^125^I]glucagon, was purchased from PerkinElmer Life Sciences. The GCGR antagonist, des-His^1^-[Glu^9^]glucagon(1–29) amide, was purchased from Tocris (Bristol, UK) and prepared as a 0.1 mm stock in water. Yeast nitrogen base and yeast extract were purchased from Difco. Flurorescein-di-β-d-glucopyranoside was purchased from Invitrogen. All other reagents were purchased from Sigma-Aldrich.

##### Constructs and DNA Manipulation

The plasmid for the generation of direct, in-frame mCherry fusion proteins was kindly donated by Steve Royale (University of Warwick). cDNA constructs of the human GLP-1R (containing an N-terminal Myc tag) and the human glucagon receptor were donated by Professor Patrick Sexton (Monash University, Australia) and Dr. Run Yu (Geffen School of Medicine at UCLA, Los Angeles, CA), respectively. Constructs for the expression of N-terminally FLAG-tagged human RAMPs were described previously (10). The cDNA construct containing a Myc-tagged CLR was provided by Dr. Michel Bouvier (University of Montreal, Canada). RAMP-GFP constructs were purchased from Cambridge Bioscience (Cambridge Bioscience Ltd., Cambridge, UK). DNA manipulations were performed using standard methods. Oligonucleotides were supplied by Invitrogen. PCR amplification used FastStart *Taq* polymerase (Roche Diagnostics, Burgess Hill, UK). All constructs were sequenced by GATC (GATC Biotech, London, UK) prior to use.

##### Cell Culture and Transfections

Dulbecco's modified Eagle's medium (DMEM) (Invitrogen) supplemented with 10% fetal calf serum and 2 mm l-glutamine was used to culture human embryonic kidney (293T) (HEK-293) cells provided by Dr. Jügen Müller (University of Warwick) in a humidified 5% CO_2_, 95% air incubator at 37 °C. Cells were transfected with Fugene 6 (Roche Diagnostics) in accordance with the manufacturer's instructions using a 1:3 (w/v) DNA/FuGENE ratio. Transfected cell lines were grown for 48 h prior to assaying. Where appropriate, pertussis toxin (PTX) (200 ng/ml) was added to ADP-ribosylate Gα_i_ for 16 h prior to assaying, thereby uncoupling receptor-mediated Gα_i_-dependent inhibition of cAMP production.

##### Analysis of Cell Surface Expression by ELISA

Cells were seeded into 24-well plates coated with poly-d-lysine and transiently transfected with receptors, RAMPs, and vector controls as appropriate. Following 48 h of growth in supplemented DMEM, medium was replaced with 3.7% formaldehyde for 15 min. Cells were washed three times with 500 μl of phosphate-buffered saline (PBS) and incubated with 1% BSA in PBS for 45 min to prevent nonspecific antibody binding. To determine receptor expression, 250 μl of primary antibody (mouse anti-Myc (Fisher), diluted 1:2500 in 1% BSA in PBS) was added for 1 h. RAMP expression was similarly assessed using an anti-FLAG M2 primary antibody (mouse (Sigma-Aldrich), diluted 1:3500 in PBS with 1% BSA). Cells were washed three times with 500 μl of PBS and reblocked (500 μl PBS + 1% BSA) for 15 min before the addition of the secondary antibody (HRP-conjugated anti-mouse IgG (GE Healthcare)) diluted 1:2500 in 1% BSA in PBS for 1 h. Following three further washes with PBS in HRP, activity was determined using SigmaFast *o*-phenylenediamine tablets (Sigma-Aldrich) according to the manufacturer's instructions. Values were normalized to Myc-GLP-1R (for receptor expression) or FLAG-RAMP2/CRLR (for RAMP expression) as 100% and cells transfected with empty vector as 0%.

##### cAMP Accumulation Assays

Transfected cells were washed in PBS, resuspended in stimulation buffer (PBS containing 0.1% BSA and 0.5 mm isobutylmethylxanthine), and seeded at 2000 cells/well in 96-well white OptiPlates. Ligands were added in the range of 1 pm to 1 mm, and cAMP accumulation was measured after 30 min of stimulation using the LANCE® cAMP detection kit (PerkinElmer Life Sciences). Values were converted to concentration using a cAMP standard curve performed in parallel.

##### Competition-binding Assays

Homologous and heterologous competition-binding assays were performed on whole cells transfected with GCGR in the absence and presence of RAMP2 using [^125^I]glucagon as the radioligand. Cells were seeded into 6-well plates and grown to 80% confluence before transfection with 1.5 μg of plasmid DNA and incubated for a further 24 h. Medium was then replaced with fresh DMEM, and cells were transferred onto a 96-well plate coated with poly-d-lysine and incubated for 24 h to achieve confluence prior to assay. Binding was performed using an adaptation of the method outlined previously (14) in a final volume of 180 μl with all components diluted in assay buffer (Krebs' HEPES buffer plus BSA, 10 mm HEPES, 4.2 mm NaHCO_3_, 11.7 mm d-glucose, 1.18 mm MgSO_4_·7H_2_O, 1.18 mm KH_2_PO_4_, 4.69 mm KCl, 118 mm NaCl, and 1.3 mm CaCl_2_·2H_2_O 0.1% BSA (w/v)). For the assay, cells were washed in assay buffer, 0.1 nm of radioligand and various concentrations of the peptides were added, and the plate was incubated for 16 h at 4 °C. Following washing with ice-cold assay buffer, 100 μl of NaOH (0.1 m) was added to each well, and the plate was incubated for 5 min on ice. Cells were removed, and the plate was washed with 100 μl of HCl (0.1 m). Radioactivity was determined in 2 ml of Safefluor scintillant (PerkinElmer Life Sciences) using a liquid scintillation counter with a count time of 3 min/sample. Values were corrected for nonspecific binding as determined by the amount of radioligand detected bound to cells not expressing GCGR.

##### Live Cell Imaging in HEK-293 Cells

Transfected cells were seeded into 8-well microscope slides (Thistle Scientific, Glasgow, UK) and incubated (37 °C; 5% CO_2_, 95% air) for 25 h. Prior to imaging, growth medium was replaced with a Hepes buffer as detailed previously (15) prewarmed to 37 °C. Cells were viewed on a Personal DeltaVision system (Applied Precision, Issaquah, WA) equipped with a photometric CoolSNAP HQ camera (Roper Scientific), and deconvolution was applied to images for visual clarity as described previously (16).

##### Yeast Strain Construction and Assay

General yeast procedures were performed as described previously (17, 18). *Saccharomyces cerevisiae* dual reporter strains expressing chimeras of the yeast GPA1, residues 1–467 (GPA1/Gα), with the five C-terminal amino acids of human Gα protein corresponding to Gα_s_ or Gα_i3_ (MMY84 and MMY89, respectively (19)) were used in this study because we have previously shown that both the GLP-1 receptor and GCGR couple to these subunits (17)). Mammalian GPCRs and RAMPs were introduced to the yeast strains under the control of the GAPDH promoter using plasmids containing either *ura3* (p426-GPD) or *leu2* (p425-GPD). Plasmids were transformed in a 1:1 ratio to enable equal expression levels of both the RAMP and receptors. Transformation was achieved using the lithium acetate/single-stranded DNA/polyethylene glycol method as described previously (20). Positive transformants were selected and maintained on medium lacking uracil and leucine. Receptor signaling was measured using the yeast growth assay (21) adapted as described previously (17). Initially, cell growth was performed in SD−URA−LEU medium at 30 °C to select for only those cells expressing both plasmids. Cells were then cultured to remove basal activity in SD−URA−LEU−HIS medium overnight at 30 °C and assayed using fluorescein-di-β-d-glucopyranoside-supplemented medium in the presence of different concentrations of ligand (0.01 nm to 100 mm). Fluorescence was measured on a TECAN Infinite M200 microplate reader (TECAN Ultra Evolution, Reading, UK).

##### Scintillation Proximity Assay (SPA) for G Protein Activation

Receptor/G protein activation profiles were determined using a scintillation proximity assay as described previously (22). Cells were grown to confluence in DMEM with GlutaMAX, supplemented with 10% FCS and 1× penicillin/streptomycin, in 5% CO_2_, 95% air at 37 °C. The cells were harvested using trypsin/EDTA (Sigma-Aldrich), washed with PBS, and resuspended in electroporation buffer (20 mm HEPES, 135 mm KCl, 2 mm MgCl_2_, 2 mm ATP, 5 mm glutathione, 0.5% (v/v) Ficoll 400 adjusted to pH 7.6 using KOH) at a concentration of ∼4 million cells in 4-mm gap electroporation cuvettes (York Biosciences, UK) before the addition of the required DNA (5 μg of receptor, 15 μg of RAMP constructs). The cells were then electroporated at 0.25 kV and 960 microfarads using a Gene Pulser (Bio-Rad, Hemel Hempstead, UK) and then cultured for 48 h. The cells were then homogenized in ice-cold PBS using a Dounce homogenizer and centrifuged at 300 × *g* for 10 min at 4 °C in a final volume of 40 ml. The supernatant was collected in a fresh tube and centrifuged at 50,000 × *g* for 25 min at 4 °C. The resulting pellet was resuspended in ice-cold SPA buffer (50 mm HEPES, 100 mm NaCl, 5 mm MgCl_2_, 0.5% (w/v) BSA, pH 7.4).

##### SPA Protocol

Concentration-response curves were constructed by incubating increasing agonist concentrations with membranes (10 μg) prepared from cells transfected with receptor and 0.1 μm GDP in HEPES buffer (100 mm NaCl, 50 mm HEPES, 5 mm MgCl_2_, 0.5% (w/v) BSA fraction V, adjusted to pH 7.5 with KOH) in a total volume of 200 μl in white OptiPlates (PerkinElmer). The assay was initiated by the addition of 0.5 nm [^35^S]GTPγS and incubated for 1 h at 35 °C. The assay was terminated by the addition of Nonidet P-40 at a final concentration of 0.3% (v/v) (Roche Diagnostics) and incubated at room temperature for 30 min on a plate shaker. A 10-μl aliquot of anti-G protein antibody (G_s_, sc-383; G_i_, sc-262) at a concentration of 60 μg/μl was then added, followed by a further 30 min at room temperature before the addition of 75 μl of anti-rabbit poly(vinyl toluene) SPA beads (PerkinElmer Life Sciences). The plate was then sealed, incubated at 4 °C for 20 h, and spun at 1300 × *g* for 10 min before reading in a TopCount scintillation counter (PerkinElmer Life Sciences). Data are shown as mean ± S.E. for three independent experiments, each performed in triplicate.

##### Data Analysis

Data were analyzed using Prism version 6.0e (GraphPad Software, San Diego, CA). EC_50_ and *E*_max_ values were obtained through fitting of a three-parameter logistic equation. Relative efficacy (log τ) and equilibrium dissociation constants (log *K_A_*) were generated through use of an operational model for partial agonism (23). Fluorescent image analysis was performed using ImageJ version 1.46b using the QUIMP plugin as described previously (17, 24). Statistical differences were analyzed before data were normalized, using a Student's test or one-way analysis of variance (ANOVA) with Bonferroni's or Dunnett's multiple comparisons as appropriate, and *p* < 0.05 was considered significant.

## Results

### 

#### 

##### RAMP2 Increases Glucagon Potency and Efficacy at the GCGR

**FIGURE 1.**
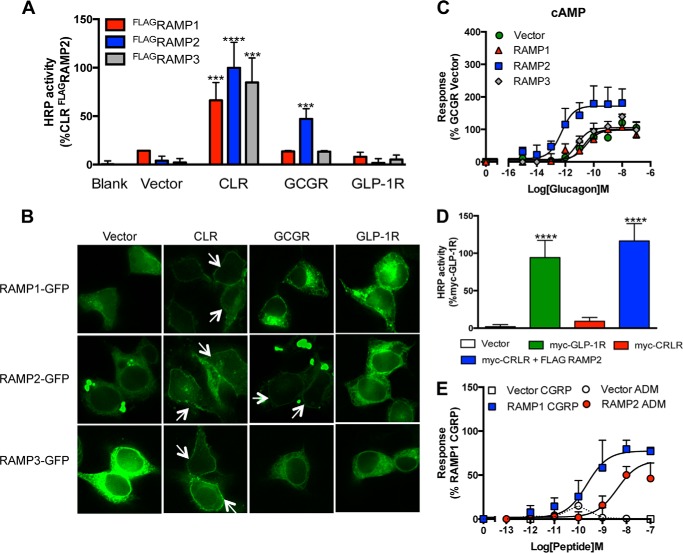
**RAMP2 interacts with the GCGR.** *A*, plasma membrane expression of ^FLAG^RAMPs in cells transfected with vector control, CLR, GCGR, or GLP-1 receptor determined by ELISA. *B*, HEK-293 cells transfected with RAMP-GFP fusion constructs in the presence and absence of various GPCRs as indicated. *Arrows*, plasma membrane localization. *C*, cAMP accumulation was determined in HEK-293 cells transiently transfected with the GCGR in the presence of vector control, RAMP1, RAMP2, or RAMP3 following 30-min stimulation with glucagon. *D*, plasma membrane expression of Myc-CLR or Myc-GLP-1R (as a positive control) determined by ELISA in HEK-293 cells co-transfected with vector control or RAMP2. *E*, cAMP accumulation, measured following 30-min stimulation of HEK-293 cells with CGRP or AM expressing CLR in the presence of vector control, RAMP1, or RAMP2. All values are the mean of at least five independent experiments ± S.E. (*error bars*). Statistical significance was determined using one-way ANOVA with Dunnett's post-test, where each data set was compared with vector control (***, *p* < 0.001; ****, *p* < 0.0001).

RAMP proteins require association with a GPCR, such as the CLR, in the endoplasmic reticulum to enable efficient translocation to the cell surface. Using an enzyme-linked immunosorbent assay (ELISA), little or no cell surface expression of the RAMPs was seen when transfected alone into HEK-293 cells. However, all three FLAG-tagged RAMPs were detected at the cell surface, upon co-transfection with the CLR (Fig. 1*A*). In contrast, the GLP-1 receptor was unable to translocate any of the RAMPs to the cell surface. Upon co-transfection of each of the RAMPs with the GCGR, only RAMP2 resulted in detectable cell surface expression (Fig. 1). Similar data were obtained using RAMP-GFP fusion constructs such that co-expression of the CLR was required to enable observation of plasma membrane-associated RAMPs, and the GCGR located only RAMP2 at the plasma membrane (Fig. 1*B*), demonstrating a positive interaction between the GCGR and RAMP2 in mammalian cells.

Like other family B GPCRs, upon ligand binding, the GCGR preferentially activates Gα_s_ to stimulate adenylate cyclase and generate cAMP. To investigate the effect of RAMP2 association on GCGR pharmacology, we constructed cAMP concentration-response curves to glucagon in HEK-293 cells transiently co-transfected with the GCGR and each of the individual RAMPs. Co-expression of RAMP2 induced a 2-fold increase in the maximal cAMP level (*E*_max_) and a 10-fold increase in the potency (EC_50_) of glucagon compared with the vector control (Fig. 1*C* and Table 1). In contrast, co-expression of either RAMP1 or RAMP3 did not influence the *E*_max_ or EC_50_ of glucagon-mediated cAMP generation (Fig. 1*B*). In order to assess whether agonist responses of the GCGR were influenced by endogenously expressed RAMPs in HEK-293 cells, we utilized the absolute requirement of the CLR receptor to interact with a RAMP to produce a functional receptor at the plasma membrane (11). In the absence of co-transfected RAMPs, we were unable to detect the CLR at the cell surface by ELISA using a Myc tag (Fig. 1*D*). Furthermore, cAMP accumulation was not observed following stimulation with either CGRP or AM (Fig. 1*E*). These data indicate that there is insufficient endogenous RAMP expression in HEK-293 cells to interact with the CLR, thus effectively providing a null background in which to study the effect of RAMP2 association with the GCGR.

**TABLE 1:**
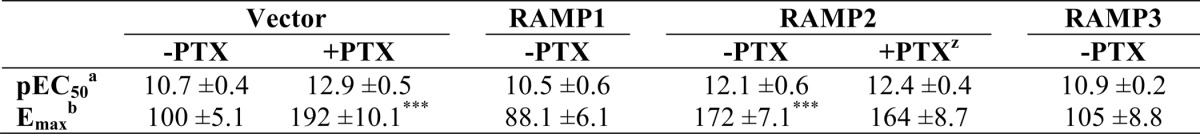
**Potency (pEC_5_) and maximal response (*E*_max_) to glucagon in cAMP assay performed on HEK-293 cells expressing the GCGR with or without the indicated RAMPs** Values were generated through fitting of a three-parameter logistic equation and represent the mean ± S.E. from 5 independent experimental repeats. Statistical significance compared with vector −PTX (RAMP2−PTX for ^z^) (***, *p* < 0.001) was determined by one-way ANOVA with Dunnett's post-test.

*^a^* The negative logarithm of the agonist concentration required to generate half the maximal response.

*^b^* The maximal response to the ligand expressed as a percentage of that obtained in the absence of pertussis toxin in cells expressing empty vector control.

##### Changes in GCGR Pharmacology Are Not Due to an Increase in Cell Surface Expression or Ligand Binding Affinity

**FIGURE 2.**
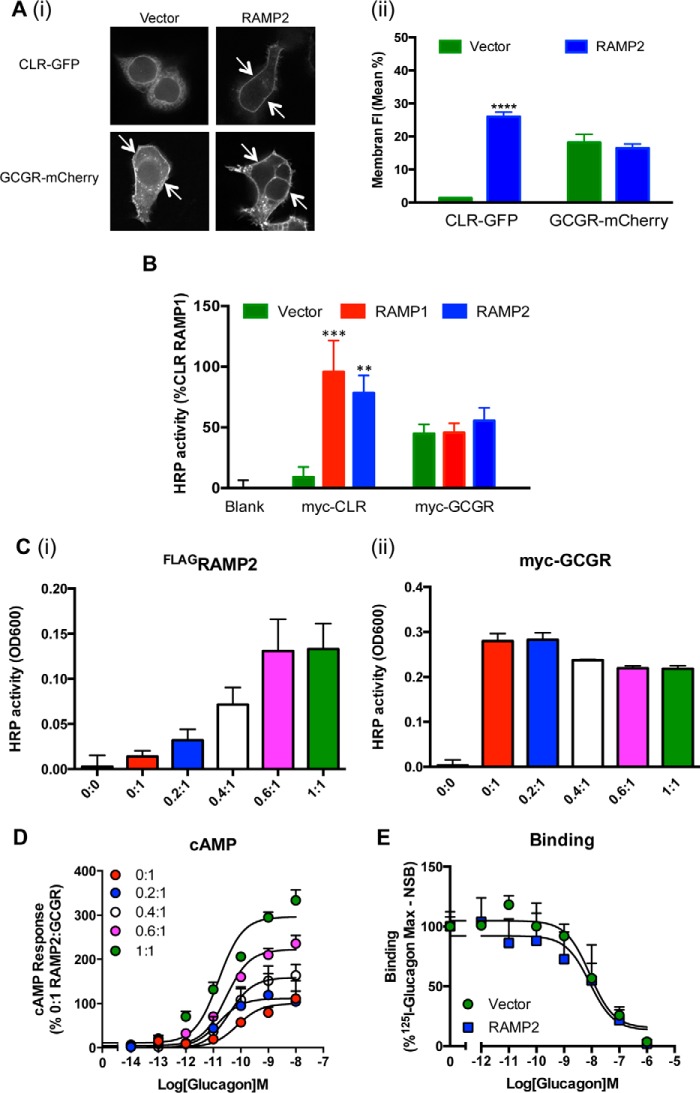
**Co-expression of RAMP2 does not affect cell surface expression of the GCGR or glucagon binding.** *A* (*i*), HEK-293 cells were transfected with fluorescently labeled receptors in the presence or absence of RAMP2. *Arrows*, plasma membrane localization. *A* (*ii*), the percentage of plasma membrane fluorescence was quantified using the QUIMP plugin for ImageJ for at least 20 cells in each population, and mean data ± S.E. (*error bars*) are represented in the bar chart. *B*, plasma membrane expression of Myc-tagged-GCGR in cells transfected with vector control, RAMP1, or RAMP2 determined by ELISA. *C*, plasma membrane expression of FLAG-tagged RAMP2 (*i*) and Myc-tagged-GCGR (*ii*) determined by ELISA in HEK-293 cells transfected with the indicated ratios of FLAG-tagged RAMP2 to Myc-tagged GCGR. *D*, HEK-293 cells expressing the GCGR and the indicated ratios of RAMP2 were assayed for cAMP accumulation following 30-min stimulation with glucagon. *E*, whole cell competition binding analysis of glucagon at the GCGR with [^125^I]glucagon tracer in the presence or absence of RAMP2. Values were corrected for nonspecific binding (*NSB*) and normalized to maximum [^125^I]glucagon binding. All values are the mean of at least five independent experiments ± S.E. Statistical significance was determined using one-way ANOVA with Dunnett's post-test where each data set was compared with vector control (**, *p* < 0.01; ***, *p* < 0.001; ****, *p* < 0.0001).

The cell surface expression and ligand binding properties of several family B receptors (10, 11) are enhanced through association with RAMPs. We used both quantitative image analysis of C-terminal receptor-fluorescent protein fusion constructs (Fig. 2*A*) and cell surface ELISAs of Myc-tagged receptors (Fig. 2*B*) to determine the effect of RAMP2 on receptor trafficking. In contrast to the CLR, which was only observed (Fig. 2*A*, *arrows*) or detected (Fig. 2*B*) at the plasma membrane when co-transfected with a RAMP, no change in the level of GCGR at the cell surface could be determined. Increases in the amount of RAMP2 relative to GCGR transfected into HEK-293 cells resulted in an elevated level of FLAG-tagged RAMP2 at the plasma membrane but did not enhance the level of Myc-GCGR detected (Fig. 2*C*), although a RAMP2-dependent increase in both potency and *E*_max_ was observed as measured through glucagon-stimulated cAMP (Fig. 2*D*). Further, a homologous competition-binding assay on cells expressing the GCGR in the presence and absence of RAMP2 revealed no change in receptor affinity for glucagon (Fig. 2*E*). These data suggest that the elevated cAMP production and increased glucagon potency observed upon association of the GCGR with RAMP2 (Fig. 1*B*) do not arise due to changes in ligand binding affinities or enhanced cell surface expression.

##### Modulation of GCGR Pharmacology by RAMP2 Is G Protein-dependent

**FIGURE 3.**
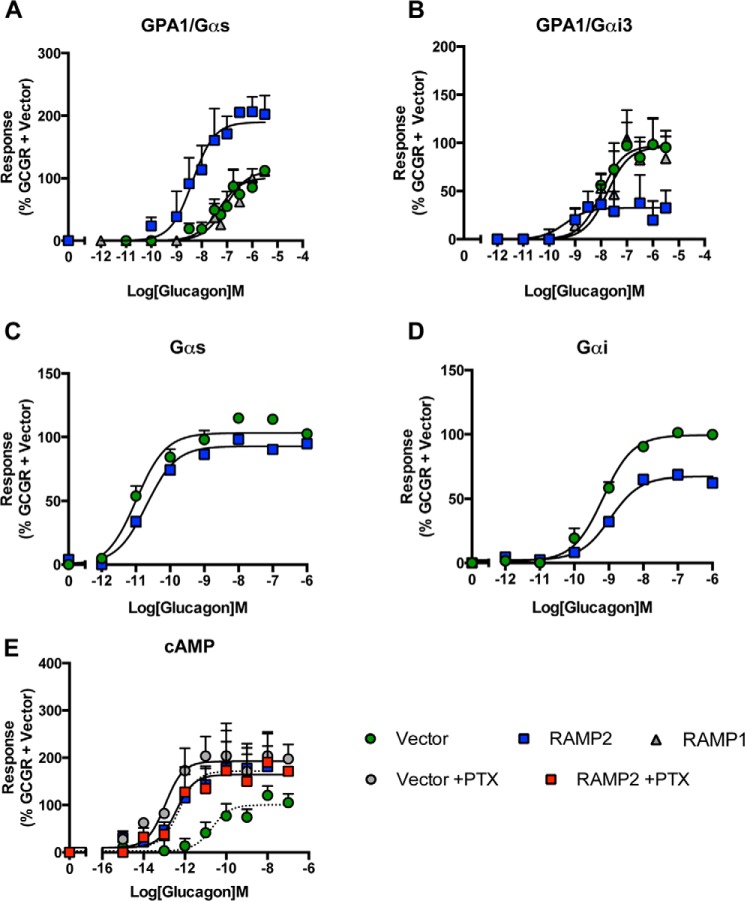
**RAMP2-mediated modulation of the GCGR is G protein-dependent.** *A* and *B*, glucagon concentration-response curves in yeast strains containing either GPA1/Gα_s_ (*A*) or GPA1/Gα_i_ (*B*) chimeras expressing the GCGR in the presence of a vector control, RAMP1, or RAMP2. Activation of the reporter gene was calculated as a percentage of the maximum response observed in the absence of RAMP2. *C* and *D*, SPAs to determine Gα_s_ (*C*) and Gα_i_ (*D*) activation upon stimulation of cell membranes, containing the GCGR in the presence or absence of RAMP2, with increasing concentrations of glucagon. *E*, HEK-293 cells containing the GCGR and RAMP2 or a vector control were incubated with 200 ng/ml PTX or a vehicle control for 16 h before stimulation with glucagon and determination of cAMP accumulation. All values are the mean of at least three independent experiments ± S.E. (*error bars*).

Intracellular levels of cAMP can be regulated by GPCRs both positively (most notably via activation of Gα_s_) and negatively (via stimulation of Gα_i_). The GCGR couples to both of these competing G protein subunits in mammalian cells (25). Further, expression of the GCGR in a yeast system containing chimeric (yeast-human) G proteins that allows the isolation of individual GPCR-G protein interactions has revealed functional coupling of the receptor to both Gα_s_ and Gα_i_ subunits (17). The chimeric G proteins in this *S. cerevisiae* system allow the coupling of human GPCRs to the endogenous yeast-mating pathway. This pathway has been modified to include a growth reporter and thereby provides the opportunity to analyze the proportion of a receptor's response attributable to individual pathways (18, 26, 27) by assessing the extent of reporter activity following stimulation with a range of ligands. Co-expression of RAMP2 (but not RAMP1) with the GCGR increased the potency and maximal response to glucagon when the receptor coupled to the chimeric protein of the yeast α-subunit and mammalian Gα_s_ (GPA1/Gα_s_) (Fig. 3*A*). However, a reduction in response was observed in strains expressing GPA1/Gα_i_ (Fig. 3*B* and Table 2). Using an SPA, we determined the activation of specific G proteins in transfected mammalian cells (Fig. 3, *C* and *D*). RAMP2 co-expression resulted in no change in glucagon-stimulated Gα_s_ activation; however, the maximal level of Gα_i_ activation achieved was reduced 2-fold (Table 2). These results demonstrate that RAMP2 expression in mammalian cells results in a change to the GCGR-mediated activation of individual G proteins similar to that seen in the yeast assay. Taken together, these data demonstrate that the ability of RAMP2 to alter glucagon-mediated responses at the GCGR is G protein-dependent; a RAMP2 interaction with GCGR appears to specifically reduce Gα_i_ activation.

**TABLE 2:**
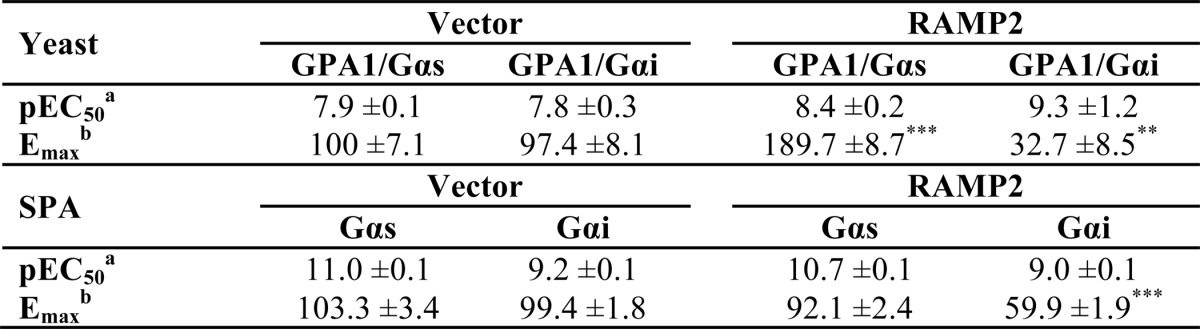
**Potency (pEC_50_) and maximal response (*E*_max_) to glucagon at the GCGR expressed with or without RAMP2 measured in yeast cell and scintillation proximity assays** Values were generated through fitting of a three-parameter logistic equation and represent the mean ± S.E. from at least 3 independent experimental repeats. Statistical significance compared with vector (**, *p* < 0.01; ***, *p* < 0.001) was determined by one-way ANOVA with Dunnett's post-test.

*^a^* The negative logarithm of the agonist concentration required to generate half the maximal response.

*^b^* The maximal response to the ligand expressed as a percentage of that obtained in the absence of RAMP expression.

Because Gα_s_ and Gα_i_ have opposing influence on adenylate cyclase activity in mammalian cells, these observations could explain the RAMP2-dependent elevation in glucagon-induced cAMP generation in HEK-293 cells (Fig. 2*B*). RAMP2 may selectively reduce coupling of the GCGR to Gα_i_, thereby leaving activation of Gα_s_ unopposed. To assess this, we pretreated HEK-293 cells with PTX to prevent receptor-mediated Gα_i_ activation (28). In cells treated with PTX, the potency and *E*_max_ of glucagon-stimulated cAMP generation by the GCGR were increased similar to those in cells co-expressing RAMP2 (but not PTX-treated) (Fig. 3*E*). PTX treatment had no effect on the potency or maximal response in cells co-expressing the GCGR and RAMP2 (Fig. 3*E* and Table 1). These data suggest that RAMP2 uncouples the GCGR from Gα_i_; this is entirely consistent with the data from the yeast cell and SPA assays.

##### RAMP2 Modulates GCGR Pharmacology in a Ligand-dependent Manner

**FIGURE 4.**
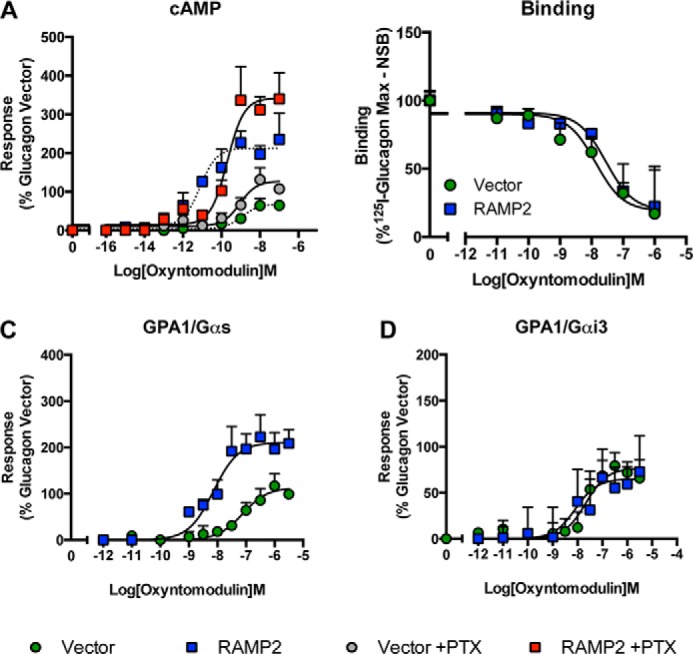
**RAMP2-mediated modulation of the GCGR is ligand-dependent.** *A*, HEK-293 cells co-transfected with the GCGR and either a vector control or RAMP2 were incubated with 200 ng/ml PTX (or vehicle control) for 16 h before stimulation with oxyntomodulin for 30 min and measurement of cAMP accumulation. *B*, whole cell competition binding analysis of oxyntomodulin with [^125^I]glucagon tracer at the GCGR in the presence or absence of RAMP2. Values were corrected for nonspecific binding (*NSB*) and normalized to maximum [^125^I]glucagon binding. GPA1/Gα_s_ (*C*) or GPA1/Gα_i_ (*D*) chimeric yeast strains transformed with the GCGR and either a vector control or RAMP2 were stimulated with oxyntomodulin, and receptor activity was determined. All values are expressed as a percentage of the maximum response observed to glucagon in the absence of RAMP2 and are the mean of at least five independent experiments ± S.E. (*error bars*).

To further investigate the effect of RAMP2 on GCGR pharmacology, we next analyzed changes in signaling in response to the related glucagon ligand, oxyntomodulin. In HEK-293 cells, challenge of the GCGR with oxyntomodulin resulted in a concentration-dependent increase in cAMP but with weaker potency than glucagon (Fig. 4*A* and Table 3). However, similar to the effects observed with glucagon, RAMP2 co-transfection increased both the potency and maximal cAMP production without affecting the binding affinity of oxyntomodulin (Fig. 4*B*). In *S. cerevisiae*, although the co-expression of RAMP2 potentiated the response to oxyntomodulin in the GPA1/Gα_s_ yeast strain, it did not affect responses in the GPA1/Gα_i_ strain (Fig. 4, *C* and *D*). This is in contrast to the inhibitory impact of RAMP2 association on glucagon-mediated GCGR responses in the GPA1/Gα_i_-expressing yeast. Furthermore, PTX treatment of HEK-293 cells expressing the GCGR had no effect on the potency of oxyntomodulin-mediated cAMP responses, although there was a small increase in *E*_max_. Intriguingly, this increase occurred independently of RAMP2 expression (Fig. 4*A*). In RAMP2-expressing cells, there was, however, a small decrease in the potency of oxyntomodulin-induced cAMP production following incubation with PTX. These data indicate that the effect of RAMP2 on the GCGR Gα_i_ response is sensitive to the activating ligand.

**TABLE 3:**
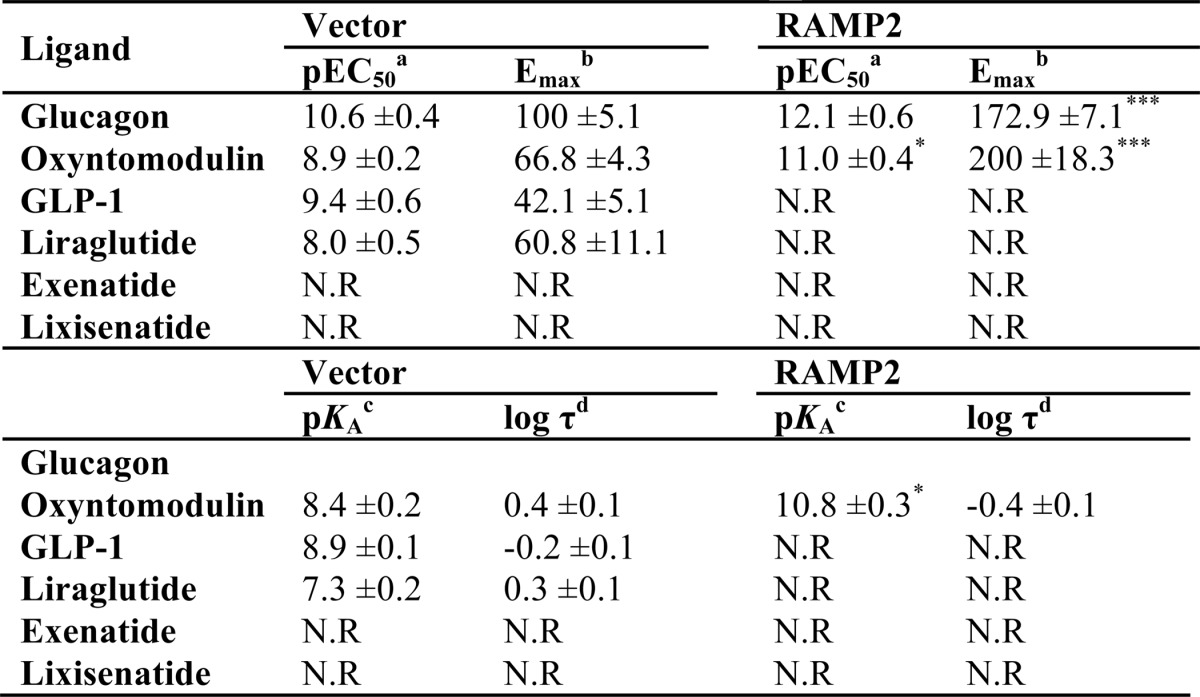
**Potency (pEC_50_), affinity (p*K_a_*), and coupling efficacy (log τ) values for various glucagon receptor agonists measured in HEK-293 cells expressing the GCGR with or without RAMP2 using a cAMP assay** All values are mean ± S.E. of five independent experimental repeats. Statistical significance compared with vector (*, *p* < 0.05, ***, *p* < 0.001) was determined by one-way ANOVA with Dunnett's post-test. N.R, no response.

*^a^* The negative logarithm of the agonist concentration required to produce a half-maximal response.

*^b^* The maximal response to the ligand expressed as a percentage of that obtained to glucagon in the absence of RAMP co-transfection.

*^c^* The negative logarithm of the relative equilibrium disassociation constant for each ligand generated through use of the operational model for partial agonism relative to glucagon.

*^d^* τ is the coupling efficiency parameter, generated by comparison with the natural ligand, glucagon, using the operational model for partial agonism.

##### GLP-1 Is a Partial Agonist at the GCGR in the Absence of RAMP2

**FIGURE 5.**
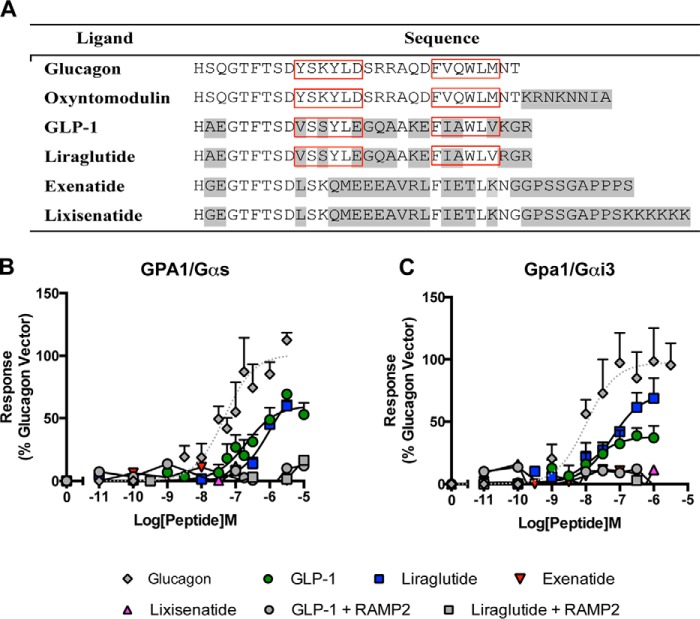
**GLP-1 receptor agonists are functional at the GCGR in the absence of RAMP2.** *A*, sequences of the various peptide ligands used in this study aligned to the natural agonist, glucagon. Amino acids differing from those in glucagon are *highlighted* in *gray. Red boxes* highlight partially conserved hydrophobic regions required for GCGR binding (31). GPA1/Gα_s_ (*B*) or GPA1/Gα_i_ (*C*) chimera yeast strains containing GCGR in the presence or absence of RAMP2 were used to determine reporter gene activity following stimulation with various GLP-1 receptor agonists. All data are expressed as a percentage of the maximum response observed to glucagon in the absence of RAMP2 and are the mean of at least five independent experiments ± S.E. (*error bars*).

Tissue-specific processing of pro-glucagon produces several peptides, including oxyntomodulin, glucagon, and GLP-1 (29), which, in its major post-prandial active form (GLP-1(7–36) amide, hereafter referred to as GLP-1), is considered to act in opposition to glucagon by stimulating insulin secretion. Although oxyntomodulin and glucagon can also stimulate the GLP-1 receptor (17, 29, 30), a limited number of studies have suggested that GLP-1 cannot bind to the GCGR (31, 32). Given that similarities between both ligands (Fig. 5*A*) and receptors (33) of this family are comparable with or greater than those in other systems (*e.g.* the calcitonin or parathyroid hormone families) that show common binding properties, we wished to determine whether a reciprocal activation of the GCGR by GLP-1 was possible. Initially, we used yeast strains expressing the GCGR and either the GPA1/Gα_s_ or GPA1/Gα_i_ chimera, and we determined that GLP-1 was able to weakly activate the GCGR, thereby acting as a partial agonist compared with glucagon (Fig. 5, *B* and *C*, and Tables 4 and 5). The observed difference in potencies between GLP-1 and glucagon is unlikely to reflect the ability of each peptide to penetrate the yeast cell wall because alternative rank orders of activity have been reported for these ligands at the GLP-1 receptor (17).

**TABLE 4:**
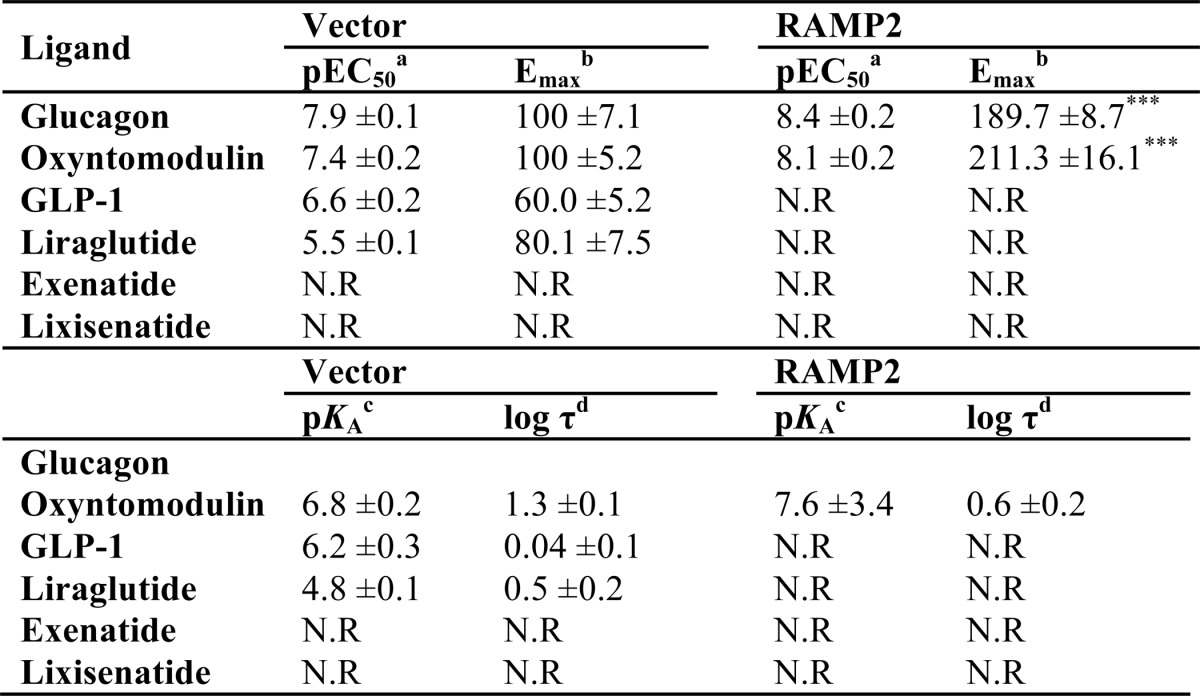
**Potency (pEC_50_), affinity (p*K_a_*), and coupling efficacy (log τ) values for various glucagon receptor agonists measured in yeast strains containing the GPA1/Gα_s_ chimera and expressing the GCGR with or without RAMP2** All values are mean ± S.E. of five independent experimental repeats. Statistical significance compared with vector (***, *p* < 0.001) was determined by one-way ANOVA with Dunnett's post-test. N.R, no response.

*^a^* The negative logarithm of the agonist concentration required to produce a half-maximal response.

*^b^* The maximal response to the ligand expressed as a percentage of that obtained to glucagon in the absence of RAMP co-expression.

*^c^* The negative logarithm of the relative equilibrium disassociation constant for each ligand generated through use of the operational model for partial agonism relative to glucagon.

*^d^* τ is the coupling efficiency parameter, generated by comparison with the natural ligand, glucagon, using the operational model for partial agonism.

**TABLE 5:**
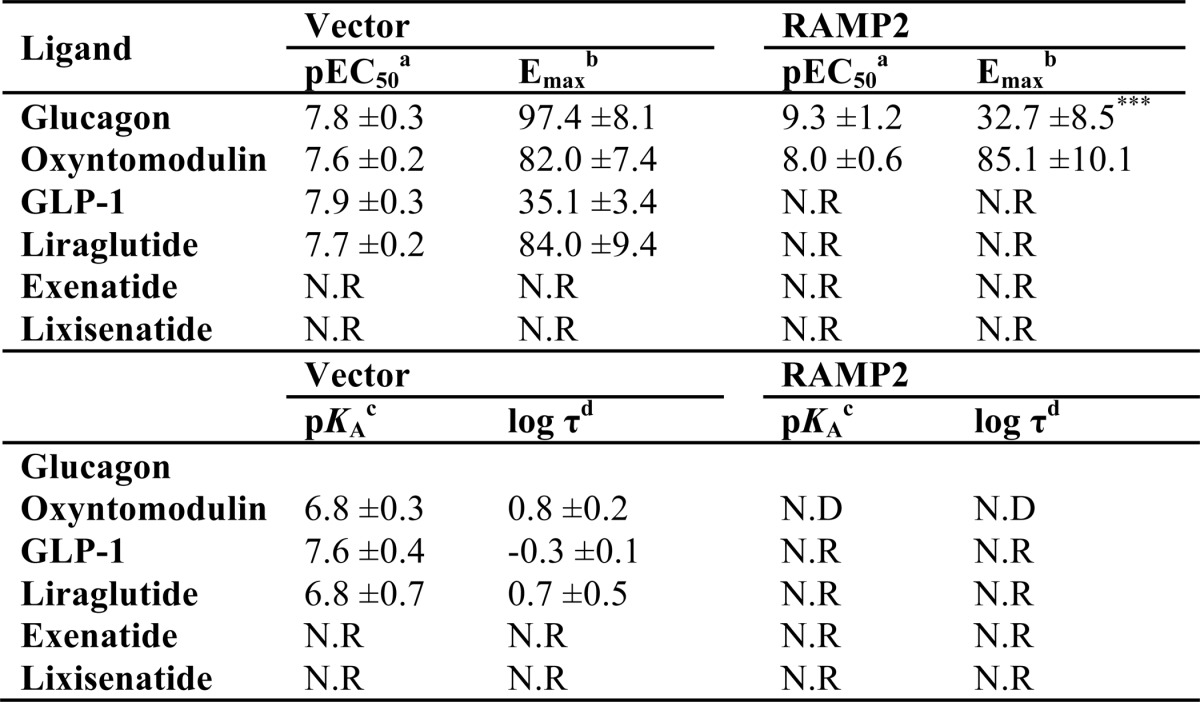
**Potency (pEC_50_), affinity (p*K_a_*), and coupling efficacy (log τ) values for various glucagon receptor agonists measured in yeast strains containing the GPA1/Gα_i_ chimera and expressing the GCGR with or without RAMP2** All values are mean ± S.E. of five independent experimental repeats. N.R, no response; N.D, not determined.

*^a^* The negative logarithm of the agonist concentration required to produce a half-maximal response.

*^b^* The maximal response to the ligand expressed as a percentage of that obtained to glucagon in the absence of RAMP co-expression.

*^c^* The negative logarithm of the relative equilibrium disassociation constant for each ligand generated through use of the operational model for partial agonism relative to glucagon.

*^d^* τ is the coupling efficiency parameter, generated by comparison with the natural ligand, glucagon, using the operational model for partial agonism.

**FIGURE 6.**
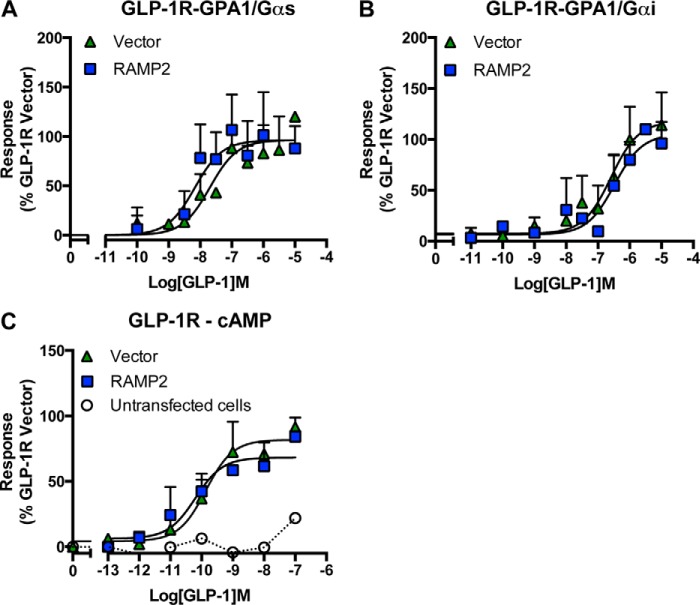
**RAMP2 co-expression does not affect GLP-1 receptor pharmacology.** The GLP-1 receptor was expressed in the presence and absence of RAMP2 in yeast strains containing GPA1/Gα_s_ (*A*) or GPA1/Gα_i_ (*B*) chimeras, and reporter gene activity was determined following stimulation with the natural agonist, GLP-1. *C*, cAMP accumulation was measured following 30-min stimulation of untransfected HEK-293 cells and those expressing the GLP-1 receptor in either the presence or absence of RAMP2. All data are expressed as a percentage of the maximum response observed to GLP-1 in the absence of RAMP2 and are the mean of at least five independent experiments ± S.E. (*error bars*).

Due to its role in insulin secretion, the GLP-1 receptor has received significant attention as a possible therapeutic target for treating type 2 diabetes, resulting in the approval of three peptide analogues of GLP-1 (liraglutide, exenatide, and lixisenatide) for clinical use. These mimetics have been designed to activate the GLP-1 receptor but also to resist proteolytic degradation, particularly by dipeptidyl peptidase IV, thereby considerably enhancing their plasma half-life beyond the 1–2 min of natural GLP-1. Significantly, among the clinically approved drugs tested here, only liraglutide (with 97% homology to GLP-1) stimulated a response in GCGR-expressing yeast strains (Fig. 5, *B* and *C*, and Tables 4 and 5). Similar to GLP-1, liraglutide displayed partial agonism at the GCGR with a reduced potency compared with glucagon. Intriguingly, co-expression of RAMP2 prevented both peptides from activating the GCGR in the yeast strains (Fig. 5, *B* and *C*). This effect was specific to the RAMP2-GCGR receptor interaction because co-expression of RAMP2 with the GLP-1 receptor had no effect on receptor pharmacology in either yeast or cAMP assays (Fig. 6).

##### RAMP2 Expression Prevents GLP-1 Binding to the GCGR

**FIGURE 7.**
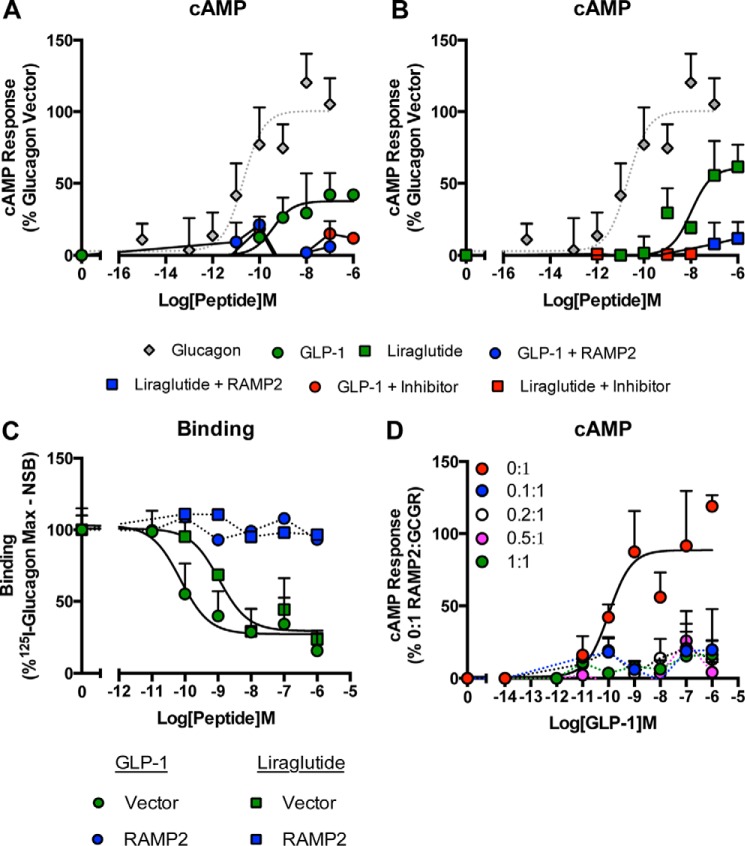
**GLP-1 specifically binds the GCGR only in the absence of RAMP2.** cAMP accumulation was measured in HEK-293 cells co-transfected with the GCGR and either a vector control or RAMP2 following stimulation with GLP-1 (*A*) and liraglutide (*B*) in the presence and absence of a GCGR antagonist, des-His^1^-[Glu^9^]glucagon(1–29) amide. *C*, whole cell competition binding analysis of GLP-1 and liraglutide with [^125^I]glucagon tracer at the GCGR in the presence or absence of RAMP2. Values were corrected for nonspecific binding (*NSB*) and normalized to maximum [^125^I]glucagon binding. *D*, HEK-293 cells expressing the GCGR and the indicated ratios of RAMP2 were assayed for cAMP accumulation following 30-min stimulation with GLP-1. All data are expressed as a percentage of the maximum response observed to glucagon in the absence of RAMP2 and are the mean of at least five independent experiments ± S.E. (*error bars*).

Having observed a RAMP2-specific modulation of GLP-1-mediated activation of the GCGR in yeast cells, we next examined these interactions in mammalian cells. Both GLP-1 and liraglutide were partial agonists of cAMP generation compared with glucagon at the GCGR (Fig. 7, *A* and *B*, and Table 3). This agonism by the GLP-1 receptor ligands was abolished upon co-transfection of RAMP2. These effects were specific to a RAMP2-GCGR interaction; RAMP2 did not affect GLP-1-mediated cAMP generation by the GLP-1 receptor in HEK-293 cells (Fig. 6*C*). Further, the addition of a GCGR antagonist (des-His^1^-[Glu^9^]glucagon(1–29) amide) inhibited GLP-1 and liraglutide signaling through the GCGR (Fig. 7, *A* and *B*). Finally, ligand binding assays revealed that, contrary to previous reports, GLP-1 and the related mimetic, liraglutide, bound to the GCGR in competition with glucagon. Significantly, both GLP-1 and liraglutide failed to bind to the GCGR upon RAMP2 co-transfection (Fig. 7*C*). Titration experiments to increase the concentration of RAMP2 revealed that the GLP-1-mediated GCGR cAMP response is extremely sensitive to the presence of RAMP2 (Fig. 7*D*). These data demonstrate that RAMP2 specifically regulates binding and thereby signal transduction of GLP-1 ligands at the GCGR. Previous work on the ability of GLP-1 receptor ligands to activate the GCGR has not considered the influence of RAMPs, which may have obscured such an interaction.

## Discussion

RAMPs have long been known to modify the pharmacology of family B GPCRs, altering the efficacy and potency of ligands and changing their ligand preference entirely, thereby having the ability to create distinct receptors (11–13). Although the interaction between RAMP2 and the GCGR was identified over 10 years ago (9), this study is the first to investigate the pharmacological consequences of the association. Here we have shown, using a combination of both mammalian and yeast cell assays, that co-expression with RAMP2 causes a marked change in GCGR pharmacology through alterations in G protein coupling that are also dependent upon the activating ligand.

One particular strength of our approach, using both the mammalian and yeast systems, is that we have identified subtle differences in signaling that might not have been apparent through the use of a single assay. For example, in HEK-293 cells, the impact of RAMP2 on cAMP responses to glucagon and oxyntomodulin was essentially the same, with a potentiation in cAMP accumulation in both cases (Figs. 3 and 4). However, in the yeast assay, in which the activation of individual (competing) G protein pathways can be assessed, there were clear ligand-dependent differences in the impact of RAMP2 on signaling by the GCGR. In the case of glucagon, RAMP2 potentiated the response mediated through the GPA1/Gα_s_ chimera (Fig. 3*A*) but reduced activation through the GPA1/Gα_i_ chimera (Fig. 3*B*). In contrast, RAMP2 modulation of the oxyntomodulin response only occurred through the GPA1/Gα_s_ chimera (Fig. 4, *A* and *B*).

In HEK-293 cells, co-expression of RAMP2 with the GCGR resulted in potentiation of both glucagon- and oxyntomodulin-stimulated cAMP. However, the GCGR couples to both stimulatory (Gα_s_) and inhibitory (Gα_i_) G proteins that combined regulate cAMP production following receptor stimulation (25). Through a variety of different techniques, we demonstrated that the mechanism by which the RAMP2 achieved this affect is both ligand- and G protein-specific. The data suggest that when glucagon is used as the ligand, the presence of RAMP2 reduces the Gα_i_ component. In contrast, the affect on the oxyntomodulin response occurs through the potentiation of the Gα_s_ activity when measured in the yeast assay. This is broadly supported in the cAMP response, with an increase in *E*_max_ being observed following PTX treatment of HEK-293 cells. However, PTX appeared to reduce the affect of RAMP2 on the potency of oxyntomodulin. This possibly indicates the presence of another competing G protein-mediated pathway (*e.g.* Gα_q_ (7) which, through the promotion of protein kinase C activation has been reported to affect PTX-sensitivity of other Gα_s_/Gα_i_-coupled family B GPCRs (34)). The elevation of intracellular calcium by GCGR activation has been relatively well described and has been demonstrated to occur in transfected HEK-293 cells via both Gα_i_ and Gα_q_-mediated pathways (7). It is somewhat surprising that little attention has been given to the ability of the GCGR to signal via Gα_i_, and our data suggest that further work is required to fully elucidate the physiological role of the Gα_i_ coupling.

The GCGR is one of a number of receptors that regulate glucose homeostasis within the body (6). Its primary role is to promote the release of glucose from the liver to maintain suitable levels of blood glucose. The opposing role is played by the related GLP-1 receptor, which, through a number of actions but primarily through the induction of insulin release, promotes the lowering of blood glucose levels following food ingestion. We (17) and others (30, 31) have previously reported the ability of the GCGR ligands to activate the GLP-1 receptor. Significant findings of the present study are the potential for GLP-1 receptor ligands to bind and stimulate the GCGR and the ability of RAMP2 to inhibit this. Both GLP-1(7–36) amide, the major active post-prandial circulating form of GLP-1, and the synthetic, clinically used peptide liraglutide displayed partial agonism at the GCGR. Intriguingly, neither lixisenatide nor exenatide, both long acting, clinically used GLP-1 mimetics, were able to stimulate the GCGR. Significantly, activation of the GCGR by the GLP-1 receptor ligands was abolished upon co-expression of RAMP2, and this was independent of the G protein present. These results suggest that unlike glucagon and oxyntomodulin, RAMP2 exerts its effects on GLP-1-mediated GCGR activation via preventing ligand binding. Given the differential expression of RAMP2 across mammalian cell lines, these results offer a possible explanation for previous studies that have been unable to observe GLP-1 (and liraglutide) activity at the GCGR (31–32). Furthermore, given that RAMPs are expressed across many different tissues (11), including many where GCGR activity has also been reported (36–37), our results may also provide insight into physiological observations. For example, RAMP2 expression in the heart has been shown to be significantly up-regulated in mouse models of heart failure (38). Given the observations by Ban *et al.* (39) that GLP-1 has a cardioprotective role acting via a receptor other than GLP-1R and our results demonstrating that RAMP2 prevents GLP-1-GCGR activity, it would be interesting to determine whether RAMP2 interacts with the GCGR to alter signal transduction in cardiomyocytes and whether this interaction is perhaps preventing the actions of GLP-1, leading to heart failure. However, no studies to date have simultaneously assessed the cellular content of both RAMP2 and GCGR.

Interestingly, the binding experiments presented in this study suggest that GLP-1 and liraglutide display a higher affinity for the GCGR than the native peptide. However, this did not translate into an increase in potency relative to glucagon when measured as cAMP production. It is possible that the high affinity binding of these ligands promotes the activation of alternative GCGR-mediated pathways with greater potency than that seen for cAMP, but further investigation is required to determine whether GLP-1 and liraglutide are truly biased ligands at the GCGR.

Indeed, other receptors for GLP-1R ligands have been suggested (39, 40). In such reports, some effects of GLP-1 ligands have been observed in the absence of the GLP-1 receptor through the use of either specific antagonists or knock-out animals. Given the results presented here and the tissue distribution of GCGR expression (36, 37), we suggest that some of these effects may be mediated via the GCGR. However, in some assay systems, the ability of GLP-1 receptor ligands to cross-react with the GCGR may have been missed due the presence of RAMP2. These data highlight the need to consider GCGR and RAMP2 expression when designing novel therapies to prevent unwanted activity. Hybrid peptides that modulate both the GCGR and GLP-1 receptor in different ways have also been suggested as potentially beneficial (41). Previously, it has been thought that such a compound would have to display agonist activity at the GLP-1 receptor while antagonizing the GCGR. However, our data reveal the possibility of using an agonist to both receptors that is biased to different downstream pathways. For example, a ligand that stimulates the GLP-1 receptor-Gα_s_ and GCGR-Gα_i_ pathways would have an effect similar to that of an agonist-antagonist combination. Due to the observed ligand and RAMP-engendered signaling bias observed, our data offer new mechanistic information that could be useful in the development of such co-agonists.

Structure-activity relationship studies for the GLP-1 receptor have suggested various important residues required in both the receptor and ligand for binding and activation (42). Many of these reported residues are also found within the TM regions of the GCGR and are required for interaction of the N terminus of the ligand (which is relatively well conserved across the ligands used in this study (Fig. 5*A*)). Given that RAMPs have been shown to modify the ligand-GPCR interaction for other receptors (12, 43), it is possible that the formation of a RAMP2-GCGR complex subtly changes the arrangement of key residues involved in GLP-1 binding, thereby preventing its interaction. It would therefore be interesting to observe how the presence of RAMP2 alters the residue arrangement of the recently published crystal structure of the GCGR (44). Initial homology models, built upon the assumption that GLP-1 binds in a similar mode to the GCGR extracellular domain as it does to the GLP-1 receptor suggest that the interaction of RAMP2 with GCGR may block GLP-1 binding. Loop 5 of the GCGR extracellular domain is shorter than its GLP-1R counterpart, having an unusual type I′ turn (45) that creates a more constrained environment for the GLP-1 C terminus. If RAMP2 bound to the GCGR in a way similar to that of the CLR (46) then the additional steric constraints in the vicinity of the GLP-1 C terminus may discourage binding. It should be noted that these conclusions are speculative and require further in depth analysis before definitive conclusions can be drawn. The two hydrophobic regions found within glucagon and oxyntomodulin required for binding to the extracellular domain of the receptor and forming interactions with the extracellular loop 1 (33) are partially conserved in the C termini of both GLP-1 and liraglutide but not in exenatide and lixisenatide (*boxed regions* in Fig. 5*A*). These observations provide a possible explanation for the lack of activity at the GCGR displayed by the latter two ligands.

The idea that ligands can influence pathway selectivity of GPCRs, termed biased agonism, has been suggested for many years (8) and is supported by the observed structural links between the ligand-binding pocket, via TM domains, to the intracellular G protein binding site (47, 48). Furthermore, it has been shown that different G protein subunits can influence the structure adopted by a GPCR, thereby influencing ligand binding (10). Additionally, Udawela *et al.* (49) have demonstrated that the different domains of RAMP proteins enable them to influence both ligand and G protein selectivity of GPCRs. Studies using the calcitonin receptor have revealed that RAMP modulation of ligand potencies can be pathway-specific. These studies show that RAMP expression has a greater affect on amylin potency when cAMP accumulation is used as a measure of receptor activity compared with when calcium mobilization is monitored demonstrating that RAMPs may have a direct effect on G protein coupling efficiency (13). These reports, combined with our own observations, suggest that there is a complex interplay between ligand, RAMP, receptor, and G protein that combine to bring about signal transduction, with each component being able to affect the binding or activity of another. Although the GCGR is expressed in the liver, where its main biological function is to counterbalance insulin actions, it is also expressed in a vast array of other tissues (50), including the lung, pancreas, and kidney. Interestingly, in mouse studies, varying levels of RAMP2 mRNA have been reported in these tissues (35). RAMP2 expression was higher in the pancreas, where GCGR stimulation enhances glucagon secretion (50), than in the liver, where receptor activity stimulates glucose release (6). Understanding how the GCGR signals within each of these tissues will enable the design of biased drugs that specifically engage the most therapeutically beneficial pathways and reduce unwanted side effects. Our data, which reveal a role for RAMP2 in determining both ligand selectivity and the downstream signaling pathways of the GCGR, highlight the critical need to consider all components present in target cells when designing new therapeutics.

## Author Contributions

C. W., J. L., N. L., K. B., G. O. R., D. J. R., M. P., and C. A. R. performed experiments. C. W., J. L., N. L., T. M. S., D. P., C. A. R., S. J. D., G. B. W., and G. L. analyzed data. C. W., T. M. S., C. A. R., G. B. W., and G. L. designed experiments. C. W. and G. L. wrote the manuscript. All authors reviewed and approved the final manuscript.

## Footnotes

3 The abbreviations used are:

GCGR: glucagon receptor
GPCR: G protein-coupled receptor
GLP-1: glucagon-like peptide-1
RAMP: receptor activity-modifying protein
CGRP: calcitonin gene-related peptide
AM: adrenomedullin
CLR: calcitonin-like-related receptor
SPA: scintillation proximity assay
GTPγS: guanosine 5′-3-*O*-(thio)triphosphate
PTX: pertussis toxin.
